# Transkingdom Interactions Between Viruses and Bacteria: Implications for Wastewater Treatment Efficiency

**DOI:** 10.1007/s12560-026-09695-1

**Published:** 2026-05-16

**Authors:** Justin C. Greaves, Christopher M. Robinson

**Affiliations:** 1 Department of Environmental and Occupational Health, School of Public Health, Indiana University, 1025 E. 7th St., Bloomington, IN 47405 USA; 2 Department of Microbiology and Immunology, Indiana University School of Medicine, 635 Barnhill Drive, Indianapolis, IN 46202 USA

## Abstract

Human enteric viruses frequently encounter diverse microbial communities in wastewater, where interactions with bacteria and other microorganisms can profoundly influence their environmental persistence and transmission. Increasing evidence demonstrates that bacteria can bind and stabilize enteric viruses, thereby enhancing thermal inactivation and prolonging infectivity. These interactions not only shape viral fate during wastewater treatment but also may affect the sensitivity and interpretation of wastewater-based epidemiology, an essential tool for public health surveillance. Yet significant knowledge gaps remain regarding the molecular mechanisms that govern bacterial-viral interactions and their roles in broader wastewater microbiomes. Together, advancing our understanding of transkingdom interactions in wastewater is essential for improving treatment processes, strengthening environmental surveillance, and reducing the risk of virus transmission through contaminated water systems.

## Introduction

Wastewater, which includes sewage and industrial effluents, plays a crucial role in sanitation, water management, and resource recovery. Wastewater contains a diverse array of microorganisms, including bacteria and viruses, which originate from human and animal waste, industrial discharges, and environmental runoff (Singh et al., 2024). These microbial contaminants pose significant risks to public health and the environment if not properly managed. Bacteria such as *Escherichia coli*, *Salmonella*, and *Vibrio cholerae* can cause gastrointestinal infections and other diseases, while viruses like norovirus, rotavirus, enteroviruses, and SARS-CoV-2 are responsible for a wide range of illnesses (Chio et al., 2024, Alby & Nachamkin, 2016). If not properly treated, wastewater can contribute to disease transmission by contaminating drinking water sources and through recreational waters. Additionally, in many regions, treated effluent discharge upstream may serve as a downstream drinking water source (de facto reuse), while decentralized, non-portable reuse applications, including irrigation, toilet flushing, and industrial reuse, may also pose exposure risks if viral contaminants are not adequately removed. Therefore, effective wastewater treatment and disinfection processes are crucial for reducing viral and bacterial loads and preventing their spread to protect public health across diverse water reuse scenerios.

In addition to preventing transmission, monitoring microbial contaminants in wastewater has become essential for early detection of disease outbreaks and understanding community health trends (Mao et al., 2020). Wastewater-based epidemiology enables early detection of pathogens, providing valuable insights into community-wide infection trends, including outbreaks of diseases such as COVID-19, influenza, and gastroininal infections (Galani et al., 2022; Lu et al., 2025; Sims & Kasprzyk-Hordern, 2020, Wang et al., 2025). Techniques such as PCR and next-generation sequencing have enhanced the accuracy and sensitivity of pathogen detection in wastewater. By analyzing wastewater samples, researchers can track the presence and concentration of specific bacteria and viruses, helping to identify emerging threats and assess the impact of public health interventions. Regular surveillance also supports public health decision-making and contributes to improving wastewater treatment strategies, ensuring the microbial risks are effectively managed before treated water is discharged or reused.

Since viruses in wastewater pose a growing public health and environmental safety concern, it is imperative that we effectively manage wastewater to limit their transmission to the human population. Recent data indicate that enteric viral interactions with bacteria and amoebae, termed transkingdom interactions, can promote the environmental stability of these viruses, which may pose a significant challenge for wastewater management (Greaves et al., 2025; Waldman et al., 2017). Here, we review the current literature on this growing field of transkingdom interactions between microbiota and primarily human enteric viruses and offer a perspective on how these interactions may impact treatment systems for managing enteric viruses in wastewater. While the microbiota comprises viruses, bacteria, and fungi, much of the work in transkingdom interactions has focused on the impact of bacteria on viruses. However, this review will also discuss recent work showing that other components, such as amoebae, can also impact viral infections. Finally, for this review, we focus primarily on municipal wastewater systems that receive human fecal waste from residential, commercial, and healthcare settings. Although wastewater can include industrial effluents, agricultural runoff, stormwater, and animal waste, our discussion centers on wastewater substantially influenced by human fecal matter and is therefore relevant to human enteric virus transmission and wastewater-based epidemiology.

## Diversity of Enteric Viruses and Bacteria in Wastewater

### Enteric Viruses in Wastewater

Enteric viruses are a diverse group of viruses that infect the gastrointestinal tracts of humans and animals, often causing diseases such as gastroenteritis, hepatitis, and respiratory infections. Wastewater serves as a significant reservoir for enteric viruses, primarily due to fecal contamination from infected individuals. These viruses are often shed in high concentrations in feces, and some can remain infectious for weeks to months in wastewater, depending on environmental conditions such as temperature, pH, and the presence of microbial communities. Unlike bacteria, enteric viruses do not replicate in the environment but can persist for extended periods in water, sewage, and sludge. In this review, we focus primarily on human enteric viruses associated with clinical disease and public health risk, particularly those shed in feces and commonly detected in municipal wastewater. While some viruses discussed may also infect animals or circulate zoonotically, our emphasis is on viruses with established or suspected roles in human infection, environmental transmission, and wastewater surveillance.

Several types of enteric viruses from different viral families are commonly detected in wastewater (Table 1). Norovirus is the leading cause of viral gastroenteritis worldwide and can persist in wastewater even after conventional treatment processes (Carlson et al., 2024; Flannery et al., 2012). Norovirus is highly contagious and commonly transmitted through contaminated food and water. Norovirus’s environmental stability makes it a significant concern in wastewater management. Rotavirus is another enteric virus that is a major cause of severe diarrhea in infants and children. Despite vaccines, rotavirus remains prevalent in wastewater due to high fecal shedding rates among infected individuals. Hepatitis A and Hepatitis E viruses (HAV and HEV) cause viral hepatitis and are transmitted via the fecal-oral route. HAV is commonly detected in untreated and treated wastewater and can remain infectious for extended periods of time (Takuissu et al., 2023). HEV has been increasingly detected in wastewater, raising concerns about its role in environmental transmission (Cuevas-Ferrando et al., 2019). Enteroviruses, including poliovirus, Coxsackievirus, and echovirus, belong to the *Picornaviridae* family and cause a wide array of diseases, ranging from mild febrile illness to severe conditions such as meningitis and myocarditis. Poliovirus, in particular, has been extensively monitored in wastewater as part of global eradication efforts (Whitehouse et al., 2024). Adenoviruses are also highly stable in waste environments and can resist disinfection processes, making them persistent contaminants in wastewater (Jiang et al., 2006). These double-stranded DNA viruses are commonly associated with gastroininal, respiratory, and ocular infections. Finally, astroviruses are frequently detected in wastewater (Takuissu et al., 2023). These viruses cause gastroenteritis, particularly in children. Astrovirus is commonly associated with foodborne and waterborne outbreaks worldwide.

**Table 1:** Viruses found in wastewater

Virus	Family	Detection in wastewater (gc/L)*	Interactions with bacteria	Reference
Adenoviruses	Adenoviridae	10^3^ – 10^6^	*Bacillus subtilis*	(Boehm et al., 2024; Greaves et al., 2025)
Enteroviruses	Picornaviridae	10^5^ – 10^7^	Various Gram-positive and Gram-negative bacterial species	[Brinkman 2017, Kuss et al., 2011, Robinson 2014, Dhalech 2021, Erickson et al., 2018, Aguilera 2019]
Noroviruses	Caliciviridae	10^3^ - 10^4^	HBGA-expressing bacteria	(McCall et al., 2021-Miura et al., 2013)
Picobirnaviruses	Picobirnaviridae	10^3^ - 10^6^	No documented interactions	(Hamza et al., 2011)
Rotaviruses	Reoviridae	10^2^ – 10^5^	*Lactobacillus*, *Bifidobacterium*, *Bacteroides thetaiotamicron*	(Bonanno 2025, Raev et al., 2022)
Reoviruses	Reoviridae	10^3^ - 10^4^	*Bacillus subtilis*, *Escherichia coli*	(Betancourt & Gerba, 2016; Berger et al., 2017)
Hepatitis A Virus	Picornaviridae	10^4^ - 10^8^	Various *Bacillus* species, *Enterococcus faecium*, *Pseudomonas alcaligenes*	(McCall et al., 2021; Deng & Cliver, 1995)
Hepatitis E Virus	Hepeviridae	10^4^ -10^5^	No documented interactions	(Dimeglio et al., 2025)
Astroviruses	Astroviridae	10^6^ - 10^8^	*Escherichia coli*, *Enterococcus faecalis*	(Le Cann et al., 2004, Li, et al., 2024)
Sapoviruses	Caliciviridae	10^4^ - 10^6^	No documented intearactions	(McCall et al., 2021)
Polyomaviruses	Polyomaviridae	10^3^ - 10^7^	No documented interactions	(Shaheen et al., 2024)
Coronaviruses (e.g., SARS-CoV-2)	Coronaviridae	10^3^ - 10^6^	No documented studies of direct interactions, but SARS-CoV-2 spike protein has been shown to bind to LPS	(Kaya et al., 2022; Petruk et al., 2020)

*gc/L = genome copies per lit

### Sources and Pathways of Enteric Viruses in Wastewater

Enteric viruses are shed in human feces and enter wastewater treatment plants through sewer systems that collect raw sewage from residential, commercial, and healthcare sources. In areas with inadequate sanitation infrastructure, untreated sewage may be directly discharged into water bodies, increasing the risk of viral contamination. Even in well-developed wastewater treatment systems, some enteric viruses can evade removal processes and persist through treatment stages.

Enteric viruses can spread from wastewater through several pathways. First, the spread can occur through direct discharge into water bodies. Contaminated sewage released into rivers, lakes, and coastal waters can pose significant risks to human and animal health. Wastewater reuse for irrigation and agriculture may also introduce enteric viruses into crops, soil, and livestock, contributing to indirect human exposure. Similarly, treated biosolids used as fertilizer may harbor enteric viruses, which can pose a risk for potential transmission through food crops. Finally, during wastewater treatment processes, aerosols can be generated that contain viral particles, potentially exposing workers and nearby populations to airborne transmission. Therefore, understanding the prevalence and persistence of enteric viruses in wastewater is essential to minimize public health risks and enhance wastewater-based epidemiology efforts.

A particularly important downstream consequence of wastewater discharge is contamination of recreational waters, which has been repeatedly linked to viral disease outbreaks. Enteric viruses are commonly associated with outbreaks from fresh or marine recreational water, particularly when fecally contaminated waters are used for swimming or other activities. A systematic review identified over 50 recreational water-associated outbreaks worldwide caused by enteric viruses such as norovirus, hepatitis A, Coxsackievirus, echovirus, and adenovirus between 1951 and 2006 (Sinclair et al., 2009). Epidemiological surveillance in the United States between 2009 and 2019 demonstrated that 22% of enteric pathogen outbreaks associated with untreated recreational water were caused by norovirus (Vanden Esschert et al., 2020). Local studies of coastal bathing areas have also detected enteric viruses, including human adenoviruses, astroviruses, enteroviruses, and noroviruses, in swimming zones linked to sewage discharge, with seasonal patterns (Wei et al., 2020). These examples highlight the importance of monitoring enteric viruses in environmental waters and linking wastewater contamination to recreational health risks.

### Bacterial Communities in Wastewater

Wastewater harbors a vast and diverse array of bacterial communities that play a role in organic matter degradation, nutrient cycling, and the overall function of wastewater treatment systems. These bacteria originate from human feces, industrial and agricultural runoff, and other environmental sources. Bacterial communities in wastewater are highly dynamic and vary based on factors such as wastewater source, treatment stage, temperature, and nutrient availability. Understanding the composition and function of these bacterial communities is crucial for improving treatment processes, monitoring public health risks, and assessing interactions that may affect pathogen persistence, including enteric viruses. Numerous high-throughput sequencing studies and metagenomic analyses have revealed that municipal wastewater can contain many distinct bacterial taxa, reflecting substantial taxonomic richness across treatment stages. While a comprehensive description of wastewater bacterial diversity is beyond the scope of this review, across geographical regions and treatment configurations, the phyla Proteobacteria, Bacteroidetes, and Firmicutes are consistently identified as the predominant bacterial groups in wastewater (Palanisamy et al., 2022; Wu et al., 2019, Oluseyi Osunmakinde 2019, Stewart et al., 2024). Proteobacteria are often the most abundant and functionally diverse bacterial groups in wastewater. Proteobacteria include many bacterial species involved in nitrogen cycling, organic matter degradation, and biofilm formation found in wastewater. Genera such as *Escherichia/Shigella*,* Pseudomonas*, *Aeromonas*, and *Acinetobacter* are commonly detected and play roles in both beneficial wastewater treatment and pathogen persistence (Govender et al., 2021; Wu et al., 2022; Aliza et al., 2025). Further, Bacteroidetes, which include *Bacteroides* and *Enterococcus* species, are often used as indicators of fecal contamination in water quality monitoring (Rock et al., 2019). These predominantly anaerobic bacteria originate from human and animal feces. Finally, Firmicutes, such as *Enterococcus*, and spore-forming *Clostridium* species are also prevalent and may persist in wastewater and environmental waters for extended periods due to stress-resistant traits.

Other bacteria may be less abundant, but they play an important role in wastewater systems. Members of the Actinobacteria phylum can play key roles in the degradation of organic matter and the removal of nutrients in wastewater treatment processes (Barka et al., 2016). Genera such as *Corynebacterium* and *Mycobacterium* are commonly found in raw and treated wastewater. Finally, bacteria in the Cyanobacteria phylum are also commonly associated with surface waters. Cyanobacteria can proliferate in wastewater lagoons and stabilization ponds, contributing to algal blooms and affecting treatment efficiency (Romanis et al., 2023). Together, the extensive taxonomic diversity of wastewater bacterial communities creates a complex ecological landscape in which enteric viruses encounter a wide away of potential binding partners, stabilizing surfaces, and enzymatic activities that may influence their environmental persistence and transmission.

In addition to these dominant bacterial groups, hospital wastewater represents a significant source of multidrug-resistant bacteria into municipal systems. Healthcare facilities discharge bacteria belonging to the ESKAPE group (*Enterococcus faecium*, *Staphylococcus aureus*, *Klebsiella pneumoniae*, *Acinetobacter baumannii*, *Pseudomonas aeruginosa*, and *Enterobact*er species), which are frequently associated with antimicrobial resistance and healthcare-associated infections. The presence of these organisms in municipal wastewater not only complicates treatment but may also influence microbial community structure and interactions with enteric viruses within wastewater environments.

### Bacterial Communities in Surface Waters

Surface waters, including lakes, rivers, and streams, are typically home to a diverse range of bacteria, many of which are naturally occurring and play essential roles in nutrient cycling, decomposition, and ecosystem health. Common bacteria found in these environments include *Escherichia coli*, *Enterococcus*, *Pseudomonas*, and *Cyanobacteria* (Milligan et al., 2023; Jang et al., 2017, Jiang 2006). These bacteria help break down organic matter and contribute to the balance of microbial communities that support aquatic ecosystems (Chen et al., 2023). While many of these bacteria are benign or even beneficial under normal conditions, their populations can vary with environmental factors such as temperature, pH, and nutrient availability. However, the introduction of sewage contamination can significantly disrupt these bacterial communities (Li et al., 2024; Xie et al., 2022). Sewage, which contains high concentrations of human waste, chemicals, and organic matter, can introduce harmful pathogens and excess nutrients into surface waters. This often leads to increased levels of bacteria such as *E. coli*, *Enterococcus*, and *Clostridium* species, which are indicators of fecal contamination Greaves 2020. These bacteria can proliferate rapidly in the nutrient-rich environment created by sewage, especially in warm waters, leading to higher levels of pathogenic bacteria that pose health risks to humans and wildlife (Xie et al., 2022).

The change in bacterial populations due to sewage contamination can also have ecological impacts. For instance, the overabundance of certain bacteria in the wake of sewage discharge can lead to shifts in the microbial community, suppressing the natural diversity of bacteria essential to the healthy functioning of aquatic systems (Xie et al., 2022). Additionally, sewage-related nutrient overload, particularly nitrogen and phosphorus, can trigger algal blooms, which further alter the composition of bacterial communities (Zhu et al., 2023). Cyanobacteria, for example, can thrive under such conditions, leading to harmful algal blooms that not only affect water quality but can also release toxins harmful to aquatic life and humans (Igwaran et al., 2024). Moreover, prolonged sewage contamination can lead to long-term changes in the microbial ecosystem. Some bacteria may become resistant to antibiotics and other chemicals present in sewage, further complicating water treatment efforts (Li et al., 2022). When enteric viruses are present in sewage-contaminated environments, these different types of bacteria may interact with them and cause changes.

## Mechanisms of Enteric Virus Interactions with Bacteria in Water

### Bacterial Interactions with Enteric Viruses

Much of the early work to determine whether enteric viruses interact with bacteria was conducted in animal models, given their intestinal tropism and proximity to intestinal bacteria. Using antibiotic approaches in mice, the replication of poliovirus and reovirus, enteric viruses from the *Picornaviridae* and *Sedoreoviridae* families, respectively, was significantly reduced when bacteria were depleted (Kuss et al., 2011). Similarly, the persistence of mouse mammary tumor virus (MMTV), an enteric virus in the *Retroviridae* family, was abolished when bacteria were reduced with antibiotics (Kane et al., 2011). Similarly, other enteric viruses, including norovirus, Coxsackievirus B3, Aichi virus, Mengovirus, and adenovirus, have been shown to interact directly with bacterial cells or bacterial cell wall components, interactions that can enhance virion stability (Aguilera et al., 2019, Robinson et al, 2019, Greaves et al., 2025, Baldridge et al., 2015; Jones et al., 2014). Interestingly, these interactions may also extend beyond the intestine, as recent data indicate that respiratory bacteria can enhance influenza A virus (David et al., 2024, Rowe et al., 2020). These data suggest that bacteria may broadly impact eukaryotic viruses and play an important role in replication, pathogenesis, and transmission.

Many hypotheses have been proposed, broadly postulating that bacteria may have either *direct* or *indirect* effects on these viruses. While indirect effects, such as bacteria modulating ininal immunity to promote infection of enteric viruses have been demonstrated (Kane et al., 2011; Baldridge et al., 2015; Wilks et al., 2015), much of the work to date has focused on the direct effects of bacteria on viral particles. Electron microscopy and other assays have shown that bacteria can bind directly to norovirus, reovirus, poliovirus, and Coxsackievirus B3 (Dhalech et al., 2021; Erickson et al., 2018; Berger et al., 2017; Almand et al., 2017). Data suggest that this interaction is likely mediated by bacterial cell wall components, such as lipopolysaccharide (LPS) in Gram-negative bacteria and peptidoglycan in Gram-positive bacteria (Kuss et al., 2011; Robinson et al., 2014; Dhalech et al., 2021; Berger et al., 2017). In contrast, norovirus and rotavirus can interact with histo-blood group antigens (HBGAs) (Li et al., 2015; Miura et al., 2013; Tan & Jiang, 2014), which are typically expressed by ininal epithelial cells but are also found in certain bacteria. In vitro and EM data have demonstrated that human norovirus can bind directly to these HBGA-expressing bacteria (Miura et al., 2013; Jones et al., 2014). However, norovirus is not limited to binding to HBGA-expressing bacteria, as human and murine norovirus can also bind to various bacteria, including gut commensal bacteria (Almand et al., 2017, Madrigal et al., 2020), (Budicini & Pfeiffer, 2022). Overall, while bacteria may indirectly promote enteric viral infections by affecting the host, current data suggest that bacteria can directly interact with virions through binding.

### Bacteria-Mediated Enhancements in Enteric Virus Stability

Recent mechanistic studies have begun to elucidate the specific molecular interactions through which bacteria directly enhance enteric virus pathogenesis. A growing body of evidence demonstrates that interactions between enteric viruses and bacterial components are crucial for viral stability. Data indicate that upon binding directly to bacterial cell wall components, many enteric viral capsids are stabilized, protecting them from thermal inactivation (Robinson & Pfeiffer, 2014, Robledo 2023). For poliovirus, binding to LPS and peptidoglycan can increase its stability, thereby reducing premature RNA release (Robinson et al., 2014). Similar results were observed with reovirus, Coxsackievirus, murine norovirus, and adenovirus (Dhalech et al., 2021; Berger et al., 2017, Greaves et al., 2025, Budicini & Pfeiffer, 2022). Further, data indicate that viral interactions with bacterial cell wall components limit the efficacy of disinfectants such as bleach (Aguilera et al., 2019). Finally, data from poliovirus also suggests that this interaction can directly play a functional role in the environment. Using a mutant poliovirus with reduced binding to bacterial LPS, demonstrated that the mutant virus incurs a fitness cost in the environment following multiple rounds of infections in mice (Robinson et al., 2014). These data indicate that, by binding to bacterial cell wall components, enteric viruses may enhance their stability and thereby increase transmission to a new host. Overall, these stabilizing interactions may be conserved and may also have important implications for wastewater systems, where abundant bacteria and bacterial cell wall components could prolong virion integrity and influence environmental persistence and the detectability of enteric viruses in wastewater surveillance.

### Bacterial-Mediated Viral Inactivation and Antiviral Mechanisms

While much of the literature has focused on stabilizing interactions between bacteria and enteric viruses, leading to increased viral infectivity, certain bacterial species and their extracellular products can also exert antiviral effects. Several studies have demonstrated that bacterial proteases, including serine proteases and matrix metalloproteases, can degrade viral capsid proteins, resulting in a loss of infectivity (Corre et al., 2022). Additionally, bacterial metabolites such as lipopeptide biosurfactants may alter viral surface charge and destabilize virions (Crovella et al., 2022; Hoste et al., 2024, Johnson et al., 2019). Furthermore, competitive absorption to bacterial surfaces may also reduce viral infectivity by sequestering virions in configurations that prevent productive infections (Romanenko et al., 2025). These antiviral activities suggest that bacteria can both enhance and suppress viral persistence, highlighting the context-dependent nature of transkingdom interactions in wastewater systems.

## Implications for Water and Wastewater Treatment

### Enteric Virus Persistence in Wastewater Treatment Systems

**Fig. 1:**
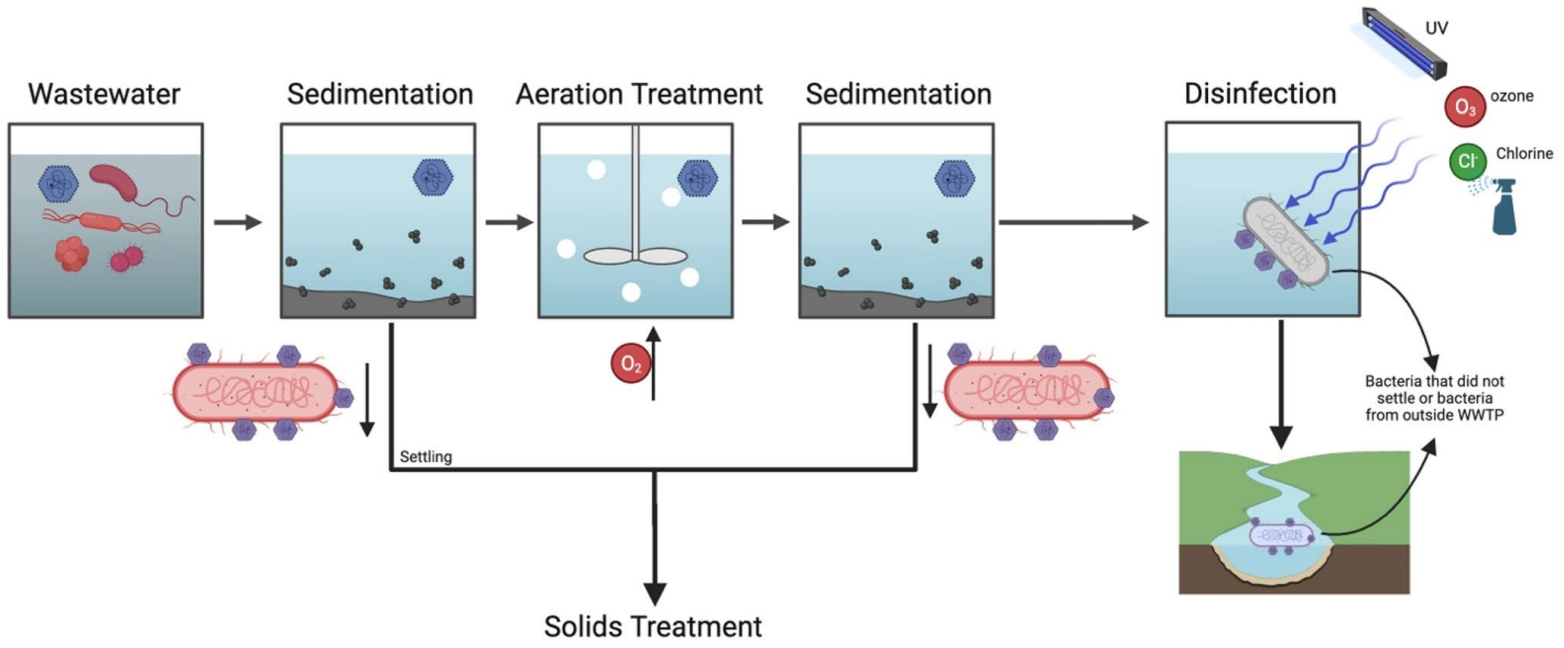
Potential pathways and fate of viruses attached to bacteria in the wastewater treatment process. Figure was created using Biorender.com

The binding of bacteria to enteric viruses can significantly influence the effectiveness of wastewater treatment processes, depending on where these interactions occur along the treatment stage. In the earlier stages, such as primary sedimentation, viruses that become adsorbed to or aggregate with bacterial cells and other suspended solids may settle out more readily, potentially improving the removal of viral particles from the water phase. Multiple studies have highlighted high solid-liquid partitioning of many different types of viruses, highlighting that most viruses will settle with particles and bacteria during the early stages of wastewater treatment or be filtered out depending on the filter size (Fig. 1) (Boehm et al., 2024; Roldan-Hernandez et al., 2024). This enhanced settling not only benefits liquid-phase treatment by reducing viral loads before secondary treatment but also concentrates viruses into the sludge and solids fraction. Therefore, treated effluent water contains significantly fewer viruses before wastewater disinfection, as they are attached to bacteria or particles. Also, as a result of sedimentation, downstream solids treatment processes, such as anaerobic digestion, composting, or thermal drying, become especially important, since these steps must effectively inactivate or destroy the concentrated viruses to prevent them from re-entering the environment when biosolids are reused or disposed of (Li 2022, Franchitti 2025). The efficiency of these solids treatment processes can be influenced by the protective effect that bacterial or organic matrices may provide to embedded viruses, sometimes making them more resistant to heat or chemical disinfection (Allingham et al., 2024). Thus, understanding how viruses bind to bacteria and partition into solids is also critical for designing comprehensive treatment strategies that address both liquid and solid streams, ultimately safeguarding public health and the environment.

While primary and secondary wastewater treatment processes can reduce viral loads, viruses often remain detectable in secondary effluent, highlighting their remarkable persistence (Prado et al., 2019, Wu et al., 2025). One contributing factor, as described previously, is the association of viruses with bacteria or bacterial components, which can significantly stabilize viruses and protect them from inactivation at high temperatures during the treatment process. Beyond thermal effects, these bacterial associations can also reduce disinfectant efficacy. also reported that aggregation with suspended solids and bacterial debris can shield viruses, such as adenovirus, from UV disinfection by physically blocking UV penetration (Templeton et al., 2008). Likewise, observed that viruses embedded within bacterial flocs show greater resistance to chlorine, likely because the disinfectant is consumed or neutralized before reaching all virus particles (Chahal et al., 2016). Together, these studies illustrate how bacterial–viral interactions not only stabilize viruses structurally but also serve as protective barriers during key disinfection steps, ultimately complicating the complete inactivation of viruses during wastewater treatment and underscoring the need for advanced monitoring and treatment strategies that account for these complex microbial relationships.

### Enteric Virus Persistence in Surface Water

Beyond wastewater treatment plants, bacterial–viral interactions can also significantly affect viral persistence once effluent is discharged into surface waters. In natural aquatic environments, viruses can adsorb to free-living bacteria, bacterial biofilms, and organic particles, which can act as physical shields against environmental stressors. For instance, demonstrated that bacterially-associated viruses exhibited prolonged survival under simulated sunlight exposure compared to free viruses, likely due to shading effects that reduce direct photoinactivation (Templeton et al., 2005). Salinity and ionic strength can further promote virus aggregation with bacterial cells and organic matter, thereby enhancing environmental persistence in surface waters (Gerba & Betancourt, 2017). Additionally, biofilms on sediments and submerged surfaces can provide microenvironments that protect viruses from temperature fluctuations, UV radiation, and predation by protozoa (Sosah et al., 2025). Together, these interactions extend viral persistence in recreational waters, sustaining potential public health risks and underscoring the need for monitoring strategies that account not only for free viral particles but also those embedded in bacterial or organic matrices.

### Potential Strategies for Enhancing Enteric Virus Removal

Various strategies can be employed to improve enteric virus removal, but few agencies have yet considered these viral-bacterial interactions during disinfection. As described here, one promising approach to enhance viral removal in wastewater treatment is to directly target bacterial–virus interactions that shield viruses from disinfection. Strategies such as applying low concentrations of surfactants or enzymes to disperse biofilms before disinfection or using advanced oxidation processes like UV/H₂O₂ that generate hydroxyl radicals capable of penetrating aggregates, have been shown to enhance viral inactivation (Jimoh et al., 2023; Kokkinos et al., 2021; Loey et al., 2025). These approaches specifically disrupt the physical barriers that protect viruses, allowing disinfectants to contact and inactivate viral particles more effectively.

Another innovative strategy involves using bioengineered microbial communities that actively promote viral degradation or irreversible adsorption. For example, research has shown that bacterial proteases can degrade viral capsids, thereby reducing viral infectivity (Corre et al., 2022, 2024). Studies like suggest designing microbial consortia that both sequester viruses onto cell surfaces and degrade them enzymatically, effectively reducing viral concentrations before they reach disinfection stages (Zhu et al., 2024). The application of bacteriophages offers yet another targeted strategy: phages can infect and lyse dominant biofilm-forming bacteria, thereby disrupting biofilms that often harbor viruses (Hong et al., 2025; Chang et al., 2022). Liu et al. showed that phage treatment reduced biofilm biomass and increased the efficacy of biocides, indirectly exposing embedded viral particles (Liu et al., 2022). Similarly, reported that phage enzymes called depolymerases can degrade the extracellular polymeric substances in biofilms, improving disinfectant penetration that can reach human viruses that may be embedded in biofilms (Zurabov et al., 2023). By integrating phage therapy into treatment trains, utilities could selectively weaken bacterial barriers without resorting to higher chemical doses, which can be costly and environmentally harmful. Overall, using engineered microbial communities for biological removal complements chemical and physical processes and can maintain performance even under fluctuating environmental conditions, thereby making the system more resilient. Together, these strategies reflect a shift toward more nuanced, ecologically informed approaches to enteric virus control.

## Wastewater-Based Epidemiology (WBE) and Public Health Implications

Wastewater-based epidemiology (WBE) is a surveillance approach that uses molecular detection of pathogen-derived nucleic acids in wastewater to infer infection trends in communities. WBE has emerged as a powerful tool for monitoring enteric virus outbreaks in communities, offering an efficient, non-invasive approach to track infections across entire populations (Singh et al., 2024). WBE relies on quantitative molecular techniques such as reverse transcription quantitative PCR (RT-qPCR) and digital PCR to measure viral genome concentrations. Because many enteric viruses are excreted in feces by both symptomatic and asymptomatic individuals, they can be detected in sewage before clinical cases are reported, providing an early warning of emerging outbreaks (Singh et al., 2024, Carmo Dos Santos et al., 2024). This method captures real-time trends in viral circulation, helping public health authorities identify hotspots, monitor seasonal patterns, and assess the impact of interventions such as vaccination campaigns or sanitation improvements (Singh et al., 2024, Carmo Dos Santos et al., 2024). Furthermore, sequencing-based approaches can also be utilized to assess viral diversity, track emerging variants, and examine co-occurrence patterns. Importantly, wastewater surveillance overcomes the limitations of clinical reporting systems, which can miss mild or asymptomatic cases, making it especially valuable for tracking highly contagious or underdiagnosed enteric pathogens and protecting public health.

Beyond its use in tracking single-infection trends, WBE holds significant promise for uncovering more profound insights into the transmission dynamics of enteric viruses and their potential interactions with specific gut bacteria, as well as environmental bacteria present in sewage and wastewater treatment systems. WBE can reveal whether outbreaks of different viruses show similar temporal or spatial patterns, shedding light on shared environmental drivers or transmission pathways, such as contamination of shared water sources or surfaces (Smith et al., 2024; Haak et al., 2022). By analyzing viral genomes alongside bacterial community profiles within wastewater samples, researchers can also identify co-occurrence patterns that may suggest ecological relationships, and in the context of this review, can show whether specific bacterial taxa consistently associate with viral particles, potentially stabilizing them or enhancing their persistence (Bibby & Peccia, 2013; Rosario et al., 2009). Quantifying viral nucleic acids and microbiome profiling in WBE may provide an ecological context for interpreting viral data. Therefore, integrating microbiome data with viral surveillance in wastewater may strengthen predictive models and improve WBE results.

Critically, by continuously monitoring these ecological patterns, WBE can serve not only as a population-level infection tracker but as an early warning system capable of detecting newly emerging viral–bacterial associations that may enhance viral persistence, infectivity, or environmental survival (Saied et al., 2023). Capturing such interactions before they translate into large-scale outbreaks can significantly refine microbial risk assessments and guide targeted, proactive public health responses, such as intensified disinfection strategies, source tracking, or community-level interventions (Carmo Dos Santos et al., 2024). This integrative power of WBE extends beyond bacteria–virus relationships; it has also been demonstrated in the detection of viral–viral interactions (Tisza et al., 2023; Rodriguez et al., 2024). For example, a recent study done by showcased the co-occurrence of adenovirus and adeno-associated virus in wastewater and highlighted possible helper-virus dynamics that could influence environmental persistence and viral replication potential (Rodriguez et al., 2024). By identifying these subtle yet consequential microbial and viral networks within wastewater, WBE helps us better understand how complex ecological interactions shape the spread and evolution of enteric viruses, ultimately improving public health preparedness and environmental monitoring strategies.

## Challenges and Future Directions

Despite growing recognition of the importance of virus-microbe interactions in shaping enteric virus biology, several key challenges continue to limit our mechanistic understanding. One major obstacle is the lack of advanced molecular, structural, and imaging tools capable of visualizing virus-bacteria interactions in real time and at physiologically relevant scales. Traditional culture-based or bulk sequencing methods often miss the spatial and biochemical nuances of these interactions. Furthermore, substantial uncertainty remains regarding the contributions of specific bacterial taxa to viral persistence, environmental stability, and transmission. Identifying these relationships in complex microbial communities remains a significant technical and conceptual challenge.

Although most research to date has focused on bacterial-viral interactions, emerging evidence indicates that other microorganisms may also have similar effects. For instance, data suggest that murine norovirus can bind fungal components (Madrigal et al. 2020), suggesting that fungi may also modulate the stability of enteric viruses. Additionally, recent studies have demonstrated that viruses may also associate with and persist within free-living amoebae (Leifels et al., 2025; Schulz et al., 2025). For example, human norovirus and human adenovirus can associate with and such as *Vermamoeba vermiformis*, *Acanthamoeba polyphaga*, and *Willaertia magna*, which are commonly found in natural and engineered water systems. Viral particles have been observed within amoebal vacuoles or cytoplasm, where they remain intact for extended periods. These findings suggest that free-living amoebae could function as environmental reservoirs that protect viruses from degradation and influence their persistence and transport in wastewater systems. Expanding investigations to include fungal, protozoan, and archaeal partners will broaden our understanding of the full microbial network that shapes viral fate. Finally, a largely unexplored but equally important question concerns the reciprocal influence of viruses on microbial communities. Enteric viruses may also, directly or indirectly, impact microbiome physiology and/or composition. Understanding these bidirectional interactions will be critical to developing a comprehensive model of how microbial ecosystems affect our water systems.

Future progress will require more targeted exploration of the wastewater microbiome, an environment where enteric viruses will encounter diverse bacteria, fungi, and protists. How these communities influence viral survival, aggregation, or inactivation in wastewater remains poorly understood. Improved mechanistic understanding could guide the development of next-generation treatment strategies to mitigate bacterial-associated viral persistence. For example, disrupting stabilizing interactions between virions and bacterial cell wall components may be a promising approach. Future studies will be required to identify the best approach to disrupt this interaction and test for its viability in our water systems.

## Conclusion

Enteric virus-bacterial interactions likely play a critical role in shaping viral stability, persistence, and transmission within wastewater systems. As discussed in this review, these interactions are likely to influence the efficacy of wastewater treatment processes and ultimately impact public health. Understanding these dynamics is essential for improving wastewater treatment strategies that effectively reduce viral loads. Continued interdisciplinary research integrating microbiology, environmental engineering, and public health is urgently needed to fully elucidate these complex interactions and optimize wastewater management practices in support of disease prevention and monitoring efforts.

## Data Availability

No datasets were generated or analysed during the current study.
